# Rapid Degradation of Rhodamine B through Visible-Photocatalytic Advanced Oxidation Using Self-Degradable Natural Perylene Quinone Derivatives—Hypocrellins

**DOI:** 10.3390/bioengineering9070307

**Published:** 2022-07-11

**Authors:** Zhixian Huang, Fan Zhang, Yanbo Tang, Yongdi Wen, Zhenqiang Wu, Zhen Fang, Xiaofei Tian

**Affiliations:** 1 Guangdong Key Laboratory of Fermentation & Enzyme Engineering, School of Biology and Biological Engineering, South China University of Technology, 382 East Out Loop, University Park, Guangzhou 510006, China; 201920146659@mail.scut.edu.cn (Z.H.); 202121050731@mail.scut.edu.cn (Y.T.); 202020148989@mail.scut.edu.cn (Y.W.); btzhqwu@scut.edu.cn (Z.W.); 2 Zhuhai Institute of Modern Industrial Innovation, South China University of Technology, 8 Fushan Road, Fushan Industrial Park, Zhuhai 519100, China; 3 Xishuangbanna Tropical Botanical Garden, Chinese Academy of Sciences, Kunming 650204, China; zhangfan@xtbg.ac.cn; 4 Biomass Group, Faculty of Engineering, Nanjing Agricultural University, Nanjing 210031, China

**Keywords:** photocatalytic advanced oxidation, organic pollutants, photosensitizer, ring-opening reaction, self-degradation

## Abstract

Hypocrellins (HYPs) are natural perylene quinone derivatives from Ascomycota fungi. Based on the excellent photosensitization properties of HYPs, this work proposed a photocatalytic advanced oxidation process (PAOP) that uses HYPs to degrade rhodamine B (RhB) as a model organic pollutant. A synergistic activity of HYPs and H_2_O_2_ (0.18 mM of HYPs, 0.33% *w/v* of H_2_O_2_) was suggested, resulting in a yield of 82.4% for RhB degradation after 60 min under visible light irradiation at 470–475 nm. The principle of pseudo-first-order kinetics was used to describe the decomposition reaction with a calculated constant (k) of 0.02899 min^−1^ (R^2^ = 0.983). Light-induced self-degradation of HYPs could be activated under alkaline (pH > 7) conditions, promising HYPs as an advanced property to alleviate the current dilemma of secondary pollution by synthetic photocatalysts in the remediation of emerging organic pollutants.

## 1. Introduction

It is well known that the synthetic persistent organic pollutants (POPs) in the effluent stream from printing and dyeing operations pose a serious risk of environmental hazard and toxicity to human health. The removal of POPs from industrial water streams is always a challenge due to their high stability against natural degradation [1,2]. A significant amount of research addresses this issue through the development of techniques such as photocatalytic advanced oxidation processes (PAOP), biological degradation and adsorption, coagulation flocculation, and membrane filtration [3,4,5,6,7,8]. Among the reported methods, the coagulation flocculation, membrane filtration and adsorption methods could not completely detoxify the POPs from the environment. The biological treatment only works under milder conditions with a longer exposure time. However, PAOP has the advantages of a strong oxidizing property, fast reaction rate, free byproducts and low cost, which make PAOP one of the most promising methods applied to the remediation of POPs.

PAOP is a green technology that uses light irradiation-generated reactive oxygen species (ROS) for the oxidative degradation of organic matter, especially POPs [9]. UV/H_2_O_2_, UV/O_3_, and UV/Fe^2+^/H_2_O_2_ PAOP combine traditional Fenton and Fenton-like methods and photocatalytic technologies and use the photolysis of oxidants to generate hydroxyl radicals (OH·). OH· has exhibited good degradation activity on organic compounds with small molecular weights and simple chemical structures [10,11,12]. With an oxidation potential ranging from 1.77 to 2.74 V, OH· is considered to be the primary ROS agent that targets the POPs and responds to their degradation [11,13]. To achieve efficient catalysis of oxidants and improve the ability of the system to generate hydroxyl radicals, various metal ions with different valence states other than iron (II) have also been developed as photocatalysts [14]. In recent years, with the massive application of TiO_2_ photocatalysis, PAOP has used various types of semiconductor materials, represented by titanium dioxide and modified semiconductor materials, as photocatalysts. In the photocatalytic degradation of organic compounds mediated by semiconductor photocatalysts, the semiconductor photocatalysts are irradiated by light and excited to form electron–hole pair (EHP), which induce redox reactions in water and generate OH·. Due to their efficient generation of ROS, these photocatalysts have become one of the most compelling research hotspots in PAOP. TiO_2_ is one of the most commonly used photocatalysts in PAOP. It has the advantages of high chemical stability, high photocatalytic activity and photoinduced hydrophilicity. The degradation yield of the organic pollutant RhB, with an initial concentration of 2.08 × 10^−5^ M, reached 96% in UV-LED/TiO_2_ PAOP using a UV-LED lamp as the light source [15]. PAOP with TiO_2_ prepared by a combination of cold plasma treatment and solgel dip-coating technology could achieve 75% efficiency of RhB mineralization under a 300 min UV exposure [16]. Echavia et al. used silica gel as a carrier to immobilize TiO_2_ and measured its degradation performance for organophosphate and phosphoglycine pesticides. The system achieved 100% removal of acephate and pesticide (dimethoate) in a short time [17]. In addition to TiO_2_, inorganic photocatalysts (IPCs), such as BiVO_4_, Bi_2_WO_6_, and (RGO)-Ag, have also been developed to improve the degradation of POPs by PAOP [18,19,20]. Among them, g-C_3_N_4_/BiVO_4_ and hydrogen peroxide (H_2_O_2_) showed synergistic activities in the degradation of diclofenac sodium [20].

However, the disadvantages of IPC are still challenging their further application in PAOP. The complex procedures for the synthesis of IPC lead to high costs or unstable catalytic performance. PAOP mediated by inorganic semiconductor photocatalysts mainly depends on UV rather than visible light sources [14,21]. Moreover, the IPC suspension could not be degraded naturally and will remain in the water stream after the reaction. They face the challenge of efficient recovery for reuse to prevent secondary pollution of the treated water stream [22]. Compared with IPC, organic photocatalysts (OPC) show a greater chemical structural variety and thus promise more photocatalysis applications. In recent years, various OPC, such as fullerenes, porphyrins and organometallic frameworks (MOFs), have become a hot research spot in the field of solar photolysis of water [23,24,25,26] and photodegradation of bisphenol A [27].

**Figure 1:**
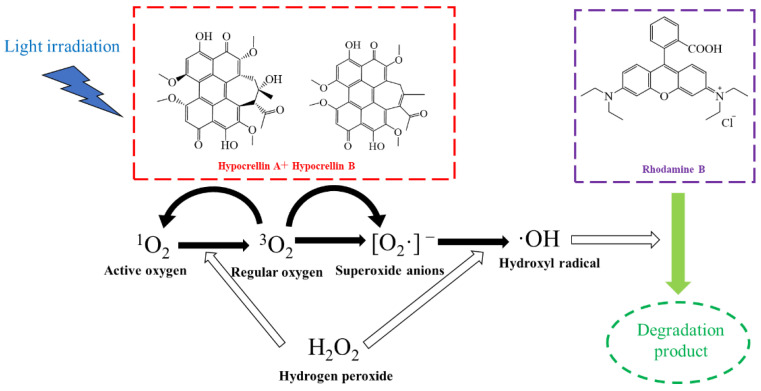
Mechanism for the generation of hydroxyl radicals by the HYPs/H_2_O_2_ PAOP.

HYPs are natural 3,10-dihydroxy-4,9-perylene-quinone derivatives that originate from the stroma of the medicinal fungus *Shiraia bambusicola* Henn. And *Hypocrella bambusae* (Berk. & Broome) Sacc. (Ascomycetes) [28,29]. HYPs generally include hypocrellin A (HA), hypocrellin B (HB), hypocrellin C, and hypocrellin D (Figure 1) with high photocatalytic activity in the generation of ROS through intense competition between type I and type II reactions [28,30,31,32,33,34,35]. Due to their broadband adsorption and sensitivity to visible light with excellent abilities to photodegrade biological molecules such as DNA and proteins [36,37,38,39,40,41], HYPs have been recognized as a promising photodynamic therapy (PDT) for the treatment of various skin diseases, such as white vulvar lesions and purpura pilaris [42,43,44,45]. As a renewable organic photosensitizer, the pH-sensitive stability of HYPs has also been reported [28].

The HYPs showed advantages of excellent photochemical but self-degrading properties. Furthermore, it was reported that the O_2_^.-^ produced by HYPs through photocatalytic reactions could be captured by H_2_O_2_, resulting in a promotion of the OH· production [20,28,46]. HYPs represent the potential to be introduced as a new photocatalyst for POP degradation through PAOP. On this basis, we propose a HYPs/H_2_O_2_ PAOP containing the photocatalyst HYPs and H_2_O_2_ to overcome the secondary pollution from artificial inorganic photocatalysts in the remediation of emerging organic pollutants. This study investigates the possibility of applying the HYPs in PAOP to form a visible-light-driven HYPs/H_2_O_2_ PAOP system and the performance of HYP/H_2_O_2_ in promoting radical hydroxyl production. The performance of HYPs/H_2_O_2_ PAOP in promoting radical hydroxyl production and organic pollutant degradation was determined by using RhB as a model compound (Figure 1). The degradation rate constants were calculated, while the degraded structures of RhB during the HYPs/H_2_O_2_ PAOP were investigated by liquid chromatography–mass spectrometry (LC/MS). As a natural perylene quinone derivative, the light-induced self-degradation of HYPs in response to changes in the pH of the surrounding aqueous solution is also discussed.

## 2. Materials and Methods

### 2.1. Chemicals

HYPs were produced by fermentation of *Shiraia bambusicola* (GDMCC 60438) described by Yan et al. [47]. After fermentation, the HYPs were extracted from the collected mycelia with dichloromethane (analytical reagent) and purified by recrystallization to a purity of 95% (with 95.7% HA and 2.9% HB, *w/w*). RhB (≥95%) and RhB standards (analytical standard, ≥97%) were purchased from Merck KgaA (Darmstadt, Germany). Methanol (HPLC grade, ≥99%) was provided by Macklin Biochemical Co., Ltd. (Shanghai, China). The H_2_O_2_ (30%, *v/v*, Fengchuan Chemical Reagent Co., Ltd., Tianjin, China), 1.3-butanediol (99%, Shanghai Aladdin Biochemical Technology Co., Ltd., China), ethyl acetate (Analytical Reagent, ≥99.5%), sodium dihydrogen phosphate (Analytical Reagent, ≥99%) and disodium hydrogen phosphate (Analytical Reagent, ≥99%, Damao Chemical Reagent Company, Tianjin, China) were all analytical grade.

### 2.2. Preparation of the HYP Aqueous Solution

HYPs (0.04 g) were mixed with 14 mL ethyl acetate and 56 mL 1,3-butanediol. The above mixed solution was stirred well and stored at 4 °C in the dark. The HYP aqueous solution was diluted to specific concentrations before use.

### 2.3. Degradation of RhB in the HYPs/H_2_O_2_ PAOP

**Figure 2:**
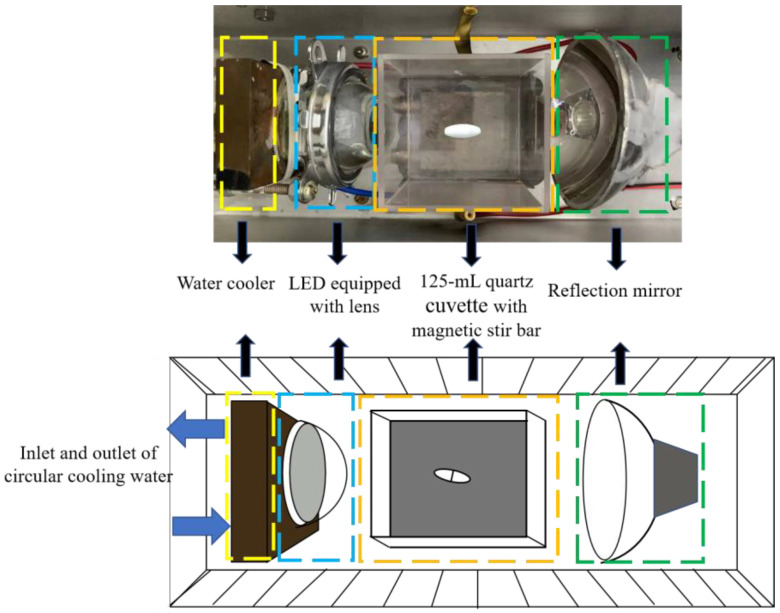
The assembly of the reactor for the photocatalytic degradation of RhB.

The HYPs/H_2_O_2_ PAOP was prepared by mixing 7.9, 15.8, 31.5, and 63.0 mL of 1.05 × 10^−3^ M HYP solutions and 1 mL of 9.9 M H_2_O_2_ in 76.1, 68.2, 52.5, and 21.0 mL phosphate-buffered solution (50 mM, pH = 7), respectively. The solution was transferred to a 125 mL (5 cm × 5 cm × 5 cm) quartz cuvette, and then 5 mL of 0.45 mM RhB solution was added to a final reaction volume of 90 mL with a RhB concentration of 2.5 × 10^−2^ mM. The mixture was irradiated with a 3.5 × 10^5^ lux LED light at 470–475 nm for 60 min (Figure 2). The sample solutions were collected at the required time. The residual RhB content was determined using an LC-20A high-performance liquid chromatograph (HPLC, Shimadzu Corporation, Kyoto, Japan) equipped with a column (Inertsil ODS-3, 5 mm, 250 × 4.6 mm, Shimadzu Corporation, Kyoto, Japan) and an SPD-20A UV detector (HPLC, Shimadzu Corporation, Kyoto, Japan). A mixture of methanol and ultrapure water (75/25, *v/v*) with a flow rate of 0.3 mL/min was used as the mobile phase. The injection volume was 20 μL, while the detector wavelength was 552 nm. The RhB concentration was calculated according to Equation (1):

(1) $$y=0.8091x\;-1.6058\;(R^{2}=0.9968)$$

The RhB degradation yield was calculated according to Equation (2):

(2) $$\mathrm{RhB}\;\mathrm{degradation}\;\mathrm{yield}\;(\%)=\frac{C_{0\;}-{\;C}_{t}}{C_{0}}\times 100\%$$

where C_0_ and C*_t_* are the RhB concentrations (M) before and after the reaction, respectively.

Since the kinetics of RhB degradation were generally assumed to follow the principle of the pseudo-first-order reaction in aqueous solution, Equation (3) was used to fit the C*_t_*-t curves during the RhB degradation in PAOP [48].

(3) $$\frac{d\;C(t)}{d\;t}=-k_{\;app}\;C(t)$$

where *t* is the reaction time (min), C is the RhB concentration (M), and *k_app_* is the apparent rate constant.

### 2.4. Determination of the Yield of Hydroxyl Radicals in the PAOP System

The generation of OH· was determined by the method based on the detection of hydroxylation products resulting from the specific reaction of salicylic acid with hydroxyl radicals [49]. A total of 10 mL of 1.05 mM HYP solution, 1 mL of 9.9 M H_2_O_2_, 5 mL of 1.7 mM salicylic acid solution was mixed with deionized water to a total volume of 100 mL. The mixture was transferred to the reactor with 3.5 × 10^5^ lux light irradiation. After 30 min, 0.5 mL of the reacting sample was collected and mixed with 2 mL of 0.04 mM FeCl_3_ solution for 30 s. The absorption at 510 nm (A510) of the mixture was determined using a 760 CRT UV–VIS spectrophotometer (INESA Analytical Instrument Co., Ltd., Shanghai, China).

The relative yield of hydroxyl radicals during PAOP was calculated using Equation (4).

(4) $$\mathrm{Relative}\;\mathrm{yield}\;\mathrm{of}\;\mathrm{the}\;\mathrm{OH}\cdot (\%)=\frac{A\;-{\;A}_{0}}{A_{H}}\;\times \;100\%$$

where A and A_0_ are the absorbance of the PAOP reaction mixture containing HYPs, HYPs/H_2_O_2_ and H_2_O_2_ before and after irradiation, respectively, and A_H_ is the absorbance of the PAOP containing only H_2_O_2_.

### 2.5. LC–MS Analysis

During the reaction, 10 mL samples were collected every 30 min. The 20 mL sample was filtered 5 times through nylon filter membranes (0.22 μm, Keyilong Lab Equipment Co., Ltd., Tianjin, China) to remove the HYPs from the solution by adsorption. Prior to analysis, the sample collected was vacuum concentrated using a RE600 rotary evaporator (Yamato Scientific Co., Ltd., Tokyo, Japan). LC/MS analysis was performed with an Acquity UPLC and Synapt G2 mass spectrometer system (Waters Corporation, MA, USA) equipped with an Acquity UPLC-BEH-C18 column (1.7 μm, 2.1 × 50 mm, Waters Corporation, MA, USA) and a SYNAPT G2 detector (Waters Corporation, MA, USA) using an electrospray ionization (ESI) source. The MS parameters were as follows: cone voltage 20 V, source temperature 120 °C, capillary voltage of 3.5 V, scanning frequency 60 Hz.

### 2.6. Determination of the Self-Degradation of HYPs

An 8 mL of HYPs (571 mg/L), 1 mL of H_2_O_2_ (30%), and 81 mL of phosphate-buffered solution (50 mM, pH = 3, 5, 7, 8 and 9) were homogeneously mixed in a 5 × 5 × 5 cm quartz cuvette. The mixture was irradiated with a 3.5 × 10^5^ lux LED light at 470–475 nm and stirred at 200 rpm for 1.5 h. After the reaction, the sample was immediately capped and stored in the dark at 4 °C prior to analysis. The absorbance of the solution was determined with a UV1800 spectrophotometer (Shimadzu Corporation, Kyoto, Japan) with a scanning range of 310 nm–710 nm.

## 3. Results and Discussion

### 3.1. Stability of the HYP Aqueous Solution

**Figure 3:**
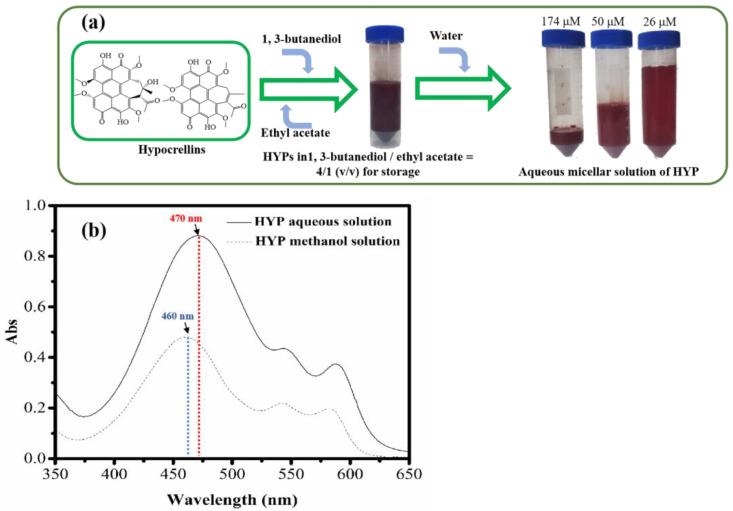
Procedure for the preparation of (**a**) aqueous micellar solution of HYPs and (**b**) absorption spectra of the HYP aqueous solution and HYPs methanol solution.

Due to the hydrophobic nature of HYPs, they are only soluble in organic solvents, such as dichloromethane, ethyl acetate and methanol. To promote the HYP distribution in water, an aqueous micellar solution of HYPs was prepared in the solvents 1,3-butanediol/ethyl acetate = 4:1 (*v/v*). After dilution with water at ratios of 1:5, 1:20 and 1:40, the micellar solution showed remarkable stability when stored for 5 days. The spectra of the HYP aqueous solution were similar to those of the HYPs-methanol solution, with the absorbance peak shifted from 460 nm to 470 nm (Figure 3). Compared to studies on improving the distribution of hydrophilic HYPs by chemical modifications [50,51,52,53], the method for preparing an HYP aqueous solution without chemical reaction was simplified with the additional advantages of (1) a 100% recovery rate derived from HYPs without purification from side reactions. (2) In contrast to toxic organic solvents such as dichloromethane, tetrahydrofuran, and N,N-dimethylformamide that can be used in chemical modification, the ethyl acetate and 1,3-butanediol required in this process are considered to be less harmful organic solvents that they are widely used and have been widely accepted in the food industry. As a classic physical modification method for hydrophobic organics, liposomes were applied to improve the water solubility of HYPs. It was reported that the preparation of Liposomal HB required high-pressure homogenization, which means that high pressure (180 MPa) and low temperature (−80 °C) were needed in the preparation of liposomal HB [54]. However, the HYP aqueous solution proposed in this work was prepared under room temperature and atmospheric pressure, lowering the demand for instruments and the cost of energy. It was reported that human serum albumin -HYP derivative nanoparticles with good water solubility were prepared through chemical modification and physical modification. During the preparation of the nanoparticles, the pH was adjusted from 11 to 7, the assembly with human serum albumin was needed for 8 h, and dialysis was needed for 48 h [55]. However, there are no time-consuming steps or pH adjustments in the procedure to prepare the HYP aqueous solution. Therefore, the HYP aqueous solution proposed in this work provides a convenient, eco-friendly, and effective way to prepare a stable water solution of HYPs for further use.

### 3.2. Improved Degradation of RhB in the HYPs/H_2_O_2_ PAOP

**Figure 4:**
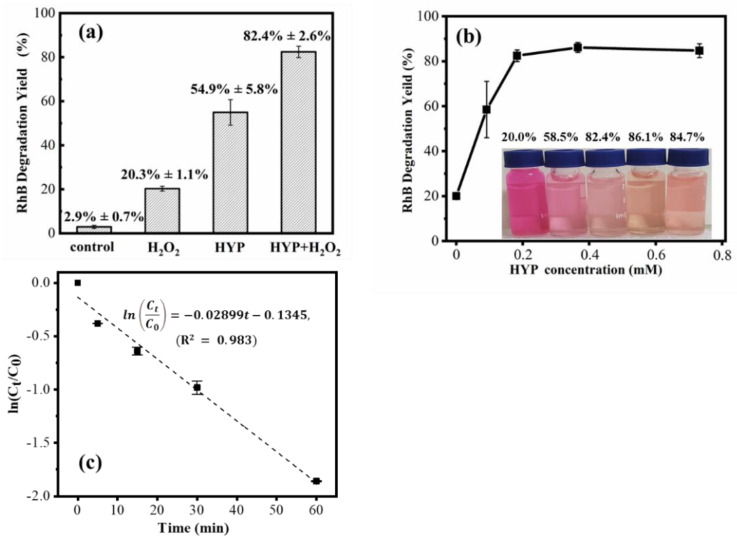
Effect of (**a**) PAOP composition and (**b**) HYPs dosage on the RhB degradation yield after a 60 min reaction. (**c**) Regression of the pseudo-first-order reaction kinetics for RhB degradation in the HYPs/H_2_O_2_ PAOP.

**Figure 5:**
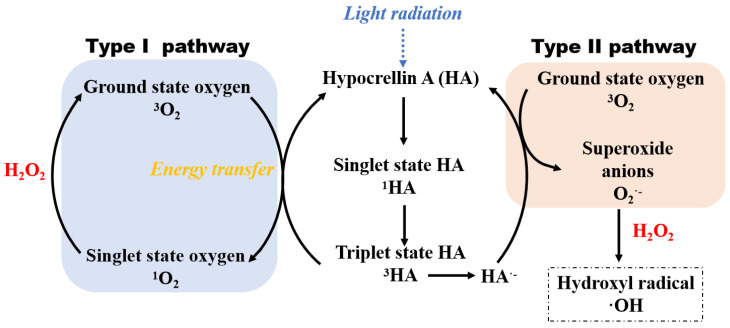
The mechanism of promotion on the generation of hydroxyl radicals in the HYPs/H_2_O_2_ PAOP using the example of the HA.

As shown in Figure 4a, the HYPs/H_2_O_2_ combination achieved an 82.4% degradation yield of RhB after a 60 min reaction, representing an improvement in degradation yield of 62.4 or 27.6% compared to H_2_O_2_ or HYPs, respectively. According to the proposed mechanism of photocatalytic generation of OH· by HYPs [28,31,32], the ground-state HYPs (^0^HYPs) could be excited by light irradiation to triplet-state HYPs (^3^HYPs) that have undergone energy transfer by reduction in water-soluble ground state oxygen (^3^O_2_) to produce singlet state oxygen (^1^O_2_) through the type I pathway and superoxide anions (O_2_^−^) through the type II pathway. It has been hypothesized that OH· exhibits higher reactivity toward RhB than toward other ROS [56,57]. The enhanced performance of HYPs/H_2_O_2_-PAOP in degrading RhB demonstrated a synergy between HYPs and H_2_O_2_ in increasing OH· production efficiency. As shown in Table 1, the calculated relative yield of OH· in HYP/H_2_O_2_ PAOP was increased by 1.04- and 1.02-fold when compared to that of HYP PAOP and H_2_O_2_ PAOP, respectively. Based on this, one of the possible mechanisms in Figure 5 could be proposed using the example of HA. Due to the introduction of H_2_O_2_, the reduction in ^1^O_2_ promoted the formation of sufficient ^3^O_2_, possibly benefiting an acceleration of O_2_^−^-production through the type II pathway. By reacting with H_2_O_2_, the increased O_2_^−^ yield could contribute to a higher OH· yield in the HYPs/H_2_O_2_-PAOP.

In the HYPs/H_2_O_2_-PAOP, the degradation yield of RhB showed a HYP-dose-dependent pattern. At the end of a 60 min reaction, the degradation yield of RhB gradually increased to 20.0%, 58.5%, 82.4%, 86.1% and 84.7% with HYP increases of 0, 50, 100, 200, and 400 mg/L, respectively (Figure 4b). A comparison of the technical performances of the HYPs/H_2_O_2_ PAOP and previously reported PAOP are shown in Table 2. It has been reported that a degradation yield of less than 80% of a 2.08 × 10^−5^ M RhB solution was obtained when 1600 mg/L TiO_2_ was under a 60 min UV exposure [15]. With excellent photocatalytic degradability to persistent organic pollutants, a dosage of 1000 mg/L Bi_2_WO_6_ led to 30% and 63% degradation yields of 1.0 × 10^−5^ M RhB after 60 and 180 min of photocatalytic reaction, respectively [19]. Similarly, a 1000 mg/L dosage of Ag/Bi_2_WO_6_ produced an 80% degradation yield of 1.0 × 10^−5^ M RhB after 60 min of photocatalytic reaction [58]. Photodegradation of 2.0 × 10^−6^ M RhB with 200 mg/L reduced graphene oxide (RGO)-Ag resulted in an approximately 70% degradation yield of RhB after a 60 min reaction [18]. The HYPs/H_2_O_2_ PAOP showed competitive activity for efficient RhB degradation compared to known inorganic photocatalysts. The silica-immobilized TiO_2_ PAOP system, which utilized solar radiation as a light source, achieved 100% degradation yields of the small-molecule organic compounds acephate and dimethoate under 105 min and 60 min of light exposure, respectively [17]. Through a synergistic effect between photo and electricity, g-C_3_N_4_/BiVO_4_-H_2_O_2_ PAOP achieved a 65% degradation yield for diclofenac sodium [20]. As RhB possesses a larger molecular weight and more complex chemical structure than acephate, dimethoate and diclofenac sodium, the HYPs/H_2_O_2_ PAOP showed more effectiveness in the treatment of POPs under visible light (Table 2).

To determine the reaction rate constant, the regression of ln (C_t_/C_0_) versus reaction times of 0, 5, 15, 30, and 60 min was performed. According to the correlation result, the calculated reaction rate constant (*k*) of the degradation reaction was 0.02899 min^−1^ (Figure 4c). The *k* for RhB degradation using the newly developed laser-cavitation method ranged from 0.00726 to 0.00937 min^−1^ [59]. The larger reaction rate constant showed an improved reaction efficiency of the HYPs/H_2_O_2_ PAOP for RhB degradation. In the UV/H_2_O_2_ PAOP, the reaction rate constants for the photocatalytic degradation of acetone and diethyl phthalate were between 0.0338–0.0666 min^−1^ and between 0.0048–0.1588 min^−1^, respectively [60,61]. The HYPs/H_2_O_2_ PAOP should also show adequate performance in the photocatalytic degradation of smaller organic compounds under visible light irradiation.

**Table 2:** The performance of the PAOP system proposed in the work and previous work.

Photocatalyst	Light Sources ^1^	Organic Pollutants	Degradation Yield (%), Initial Concentration (M)	Reaction Time (min)	References
HA	Vis	RhB	82%, 2.5 × 10^−5^	60	This study
TiO_2_	UV	RhB	96%, 2.1 × 10^−5^	180	[15]
TiO_2_ film	UV	RhB	75% ^2^, 1.0 × 10^−5^	300	[16]
Silica-TiO_2_	UV/Solar	Acephate	100%, 1.0 × 10^−4^	105	[17]
		Dimethoate	100%, 1.0 × 10^−4^	60	
ZnO	UV	Reactive black 5	72%, 1.0 × 10^−5^	780	[62]
g-C_3_N_4_/BiVO_4_	Vis	Diclofenac Sodium	65% ^3^, 3.1 × 10^−5^	180	[20]
Fe_2_O_3_/Cu_2_O(SO_4_)	UV	Acid orange 2	99%, 1.4 × 10^−4^	30	[63]
CuO/Cu_2_O	UV	Methyl orange	>90%, 2.0 × 10^−5^	30	[64]
WO_3_	UV	RhB	76%, 2.1 × 10^−6^	180	[65]
BaTiO_2_/GO	UV	Methylene blue	>80%, 1.6 × 10^−5^	120	[66]
Bi_2_WO_6_	UV	RhB	63%, 1.0 × 10^−5^	180	[19]
(RGO)-Ag	UV	RhB	70%, 2.0 × 10^−6^	60	[18]
Ag/Bi_2_WO_6_	UV	RhB	80%, 1.0 × 10^−5^	60	[58]

^1^ UV for ultraviolet light, Vis for visible light, Solar for solar light; ^2^ Percentage of mineralization determined by TOC analyze; ^3^ Synergy with hydrogen peroxide.

### 3.3. Possible Pathway for the Degradation of RhB in the HYPs/H_2_O_2_ PAOP

**Figure 6:**
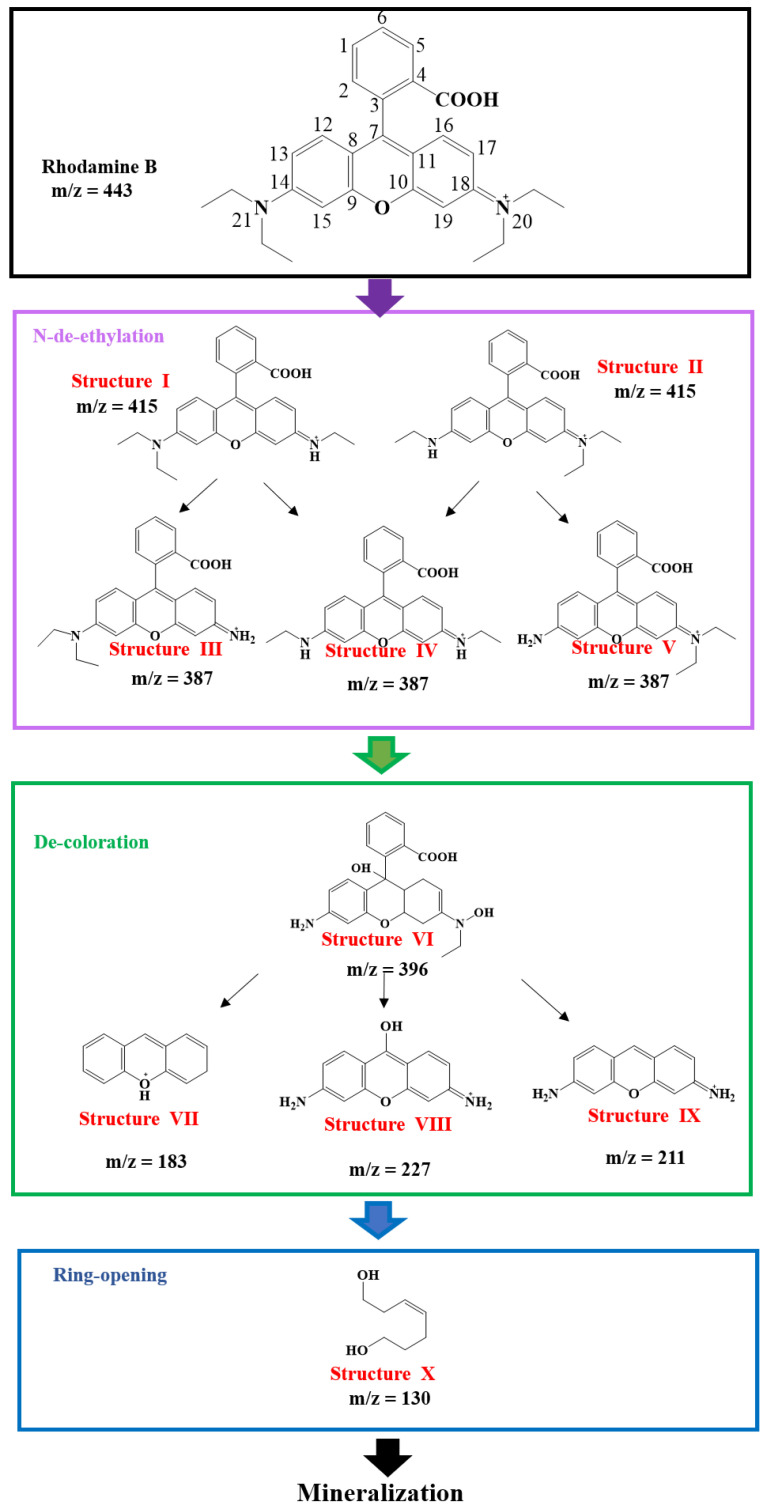
Possible pathway for the photocatalytic degradation of RhB in HYPs/H_2_O_2_ PAOP.

It was suggested that oxidation by OH· might also be involved in RhB degradation by PAOP [15]. The intermediate products during the RhB degradation were investigated by LC–MS analysis. According to the mass spectra (supporting information), 10 by-product compounds during the degradation process were identified (Figure 6) [15,67]. The LC–MS analysis also determined the existence of five intermediate structures, I, II, III, IV, and V, with the ethyl groups removed from the RhB during the decolorization reaction. The de-ethylation process is initiated by a hydroxyl radical attack on the nitrogen atoms No. 20 and No. 21, which has the highest frontline charge density of any atom in RhB, resulting in the existence of structures I, II, III, IV, and V [67]. In addition, structure VI shows hydroxyl substitution on the atom No. 20, revealing the reaction between hydroxyl radicals and nitrogen atoms. Calculation of the frontline electron density shows high reactivity from OH· to carbon atoms No. 7 [67]. Thus, the next step in the degradation of RhB is the removal of the benzoic acid group, which is considered the chromophore group in RhB. Structure VI had a hydroxyl-substituted unit on the carbon atom No. 7, showing the reaction between the carbon atom No. 7 and hydroxyl radicals during the process of benzoic acid group removal. Following the removal of the benzoic acid group, tricyclic structures VII-IX were formed in solution. Since OH· had an affinity for the carbon attached to the benzoic acid group, the released free benzoic acid group could be easily oxidized to hydroxyl-substituted structures and finally degraded in PAOP [15]. According to the number of C atoms, the hydroxyl-containing structure X was considered to be derived from the VII-IX structure through the ring opening reaction. Furthermore, mineralization may occur upon completion of the ring-opening reaction. It could be deduced that the processes of demethylation, decolorization and ring opening occur during the degradation of RhB through HYPs/H_2_O_2_ PAOP. After the completion of the ring-opening process, RhB has been continuedly decomposed into smaller fractions by the OH·.

### 3.4. The pH-Dependent Self-Degradation of HYPs

**Figure 7:**
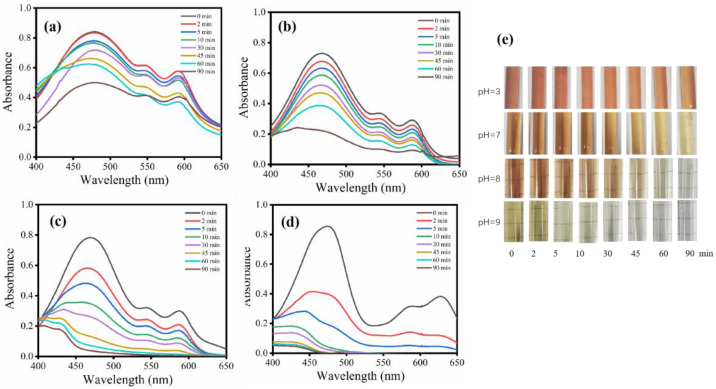
Influence of pH on the self-degradation of the HYP aqueous solution. (**a**) pH = 3, (**b**) pH = 5, (**c**) pH = 7, (**d**) pH = 8, (**e**) pH = 9, and color changes during the self-degradation of HYPs.

The self-degradation of HYPs in aqueous solution was studied at pH values of 3, 7, 8 and 9 (Figure 7a–d). Before performing light irradiation, the HYP aqueous solution with pH ≥ 7 shows a clear red color. However, it turned dark green when the pH increased from 7 to 9. The absorption spectrum shows a unique pattern with a specific absorption peak at 470 nm. This reveals the integrity of the perylene quinone structure of the HYPs. Under light irradiation, the HYP aqueous solution changed color with time in all solutions with different pH values (Figure 7e). However, the intensity of the absorption spectrum of the HYP solution showed a rapid decrease when the pH was above 7 (Figure 7c,d). The absorbance at 470 nm of HYPs at pH 9 reached almost zero, while the absorbance of HYPs under acidic or neutral conditions (pH = 3 and 7) decreased only slightly after 90 min of light irradiation (Figure 7a,b). The evidence promised the prospect of performing self-degradation of HYPs by adjusting the solution to a weakly alkaline state.

The HYPs/H_2_O_2_ PAOP is competitive with inorganic photocatalysts in terms of degradation performance and environmental friendliness. Using a pH adjustment procedure would be feasible to remove the HYPs from the aqueous solution to avoid any environmental contamination since HYPs maintain good neutral or acidic stability while being severely degraded in an alkaline environment.

## 4. Conclusions

The photocatalytic degradation of RhB through the synergistic effect between H_2_O_2_ and an organic natural photosensitizer was performed. In a 2.5 × 10^−2^ mM RhB solution, a degradation yield of 82.4% was achieved after a 60 min reaction in PAOP with 0.18 mM HYPs and 0.33% (*w/v*) H_2_O_2_. The decomposition reaction follows the principle of pseudo-first-order reaction kinetics with *k* = 0.02899 min^−1^. Owing to the property of self-degradation in a weakly alkaline environment, HYPs were an environmentally friendly photocatalyst in the PAOP of organic pollutants by alleviating a secondary pollution of the water. It was revealed that the HYPs were promising visible-light photocatalysts for application in the degradation of POPs. Further research on the effect of O_2_ concentration on the photocatalytic generation of ROS by HYPs would be meaningful for developing a high-efficiency HYPs/H_2_O_2_-PAOP. As natural and self-degradable compounds, we could prospect HYP-involved processes for environmentally friendly water photolysis and the removal of persistent toxins such as phycotoxins and aflatoxin.

## Figures and Tables

**Table 1:** The relative yield of OH· in the PAOP systems.

Reaction Mixture	Relative Yield of the OH· (%)
H_2_O_2_ PAOP	100
HYP PAOP	98.3
HYP/H_2_O_2_ PAOP	201.7

## Data Availability

Not applicable.
